# Supplementary material for: The views of UK Chinese individuals towards living and deceased-donor kidney transplantation: A qualitative interview study
Source: PLoS One. 2025 Jun 4;20(6):e0325665. doi: 10.1371/journal.pone.0325665 (PMC12136460; doi:10.1371/journal.pone.0325665)
Supplement: S1 File — (DOCX) [file pone.0325665.s001.docx]

**S1 File: Topic guide for participant interviews**

**Opener**

Welcome, introduction of researcher (and translator, if used)

Information regarding the study:

We are interested in what Chinese people living in the UK think about problems with the kidneys, and what happens when the kidneys stop working.

Participant may voluntarily start discussion, or

- Can you tell me a little bit about what you think your kidneys do and why/if they’re important?

Or

- Can you tell me a little bit about what you think happens when someone’s kidneys stop working as well as they should?

Or

- Do you or anyone you know have experience of kidney problems?

**Knowledge of kidney transplants and donation**

Do you think this is an important problem?

Where did you get information about kidney transplants/donation? (TV/Social Media/Family/friends etc).

- Positive stories?
- Negative stories (press, falung gong/ falung dafa) ?

Where would you like to see more information about kidney transplants/donation? (TV/social media)

What do you know about living kidney donation?

- Only one kidney removed?
- Can survive with one kidney
- Minimal risk long term
- Who can donate to who? Matching

Other treatments for kidney failure?

- Dialysis
- Alternative treatments

**Donating a kidney**

Would you donate a kidney if needed?

- Do you think it’s a “good”/noble thing to do

Who would you donate to/who would you not donate to? Children/Parents? More extended family?

- Respect for elders (filial piety)?
- Altruistic donation?

What would prevent you from donating a kidney?

- Religious issues
- Social/Cultural issues
- Preservation of body?
- Financial concerns
- Health concerns

What do you think would prevent other UK Chinese people from donating?

**Receiving a kidney transplant**

Would you accept a kidney transplant from a living/deceased donor if needed?

Who would you accept a kidney transplant from? Children/Parents? More extended family?

- Respect for elders (filial piety)?
- Accept a kidney from an altruistic donor?

What would prevent you from accepting an offer of a kidney transplant?

- Religious issues
- Social/Cultural issues
- Preservation of body?
- Financial concerns
- Health concerns

What do you think would prevent other UK Chinese people from accepting an offer of a kidney transplant?

**Asking about donation**

Would you feel able to ask others to consider a donating a kidney?

- What would prevent you?
- What would help you?
- Who would you ask to help if needed?
