# Supplementary material for: The views of UK Chinese individuals towards living and deceased-donor kidney transplantation: A qualitative interview study
Source: PLoS One. 2025 Jun 4;20(6):e0325665. doi: 10.1371/journal.pone.0325665 (PMC12136460; doi:10.1371/journal.pone.0325665)
Supplement: Attachment — Submitted filename: Response to comments 15_04_25.docx [file pone.0325665.s004.docx]

|  | **Comment** | **Response** |
| --- | --- | --- |
|  | Reviewer 1 |  |
| 1 | Based on the aims, the team wanted “to investigate and understand the attitudes of UK Chinese individuals towards kidney donation and transplantation.” Four concepts are being explored: being a living kidney donor, being a deceased donor, accepting a kidney from a deceased donor, accepting a kidney from a living donor. These are 4 different research questions and ethnic barriers and perceptions ought to be explored separately to truly understand the participants perceptions. For example, sense of familial duty and the need for a close immunological match may be of relevance considering being a living kidney donor and accepting a living donor, respectively. Some segregation of these concepts in the analysis will likely generate unique themes and truly allow an in depth understanding of barriers to donation and transplantation in this community, AND increase the impact of this work. | We thank Reviewer 1 for their suggestion and have attempted to be clearer about how individual themes relate to different aspects of transplantation. However, we have not presented findings under four categories as many beliefs, views, and perspectives will be relevant to all aspects of transplantation, and beliefs about organ donation are often inextricably linked to beliefs about organ acceptance e.g., the concept of reciprocity: if I’m willing to accept a transplant I should be willing to donate. We have added the following sentence to our results at line 152-155 and edited the description of each sub-theme to be more explicit.  *“We have highlighted where themes appeared to specifically relate to receiving or donating an organ or partaking in living or deceased donation. However, views and beliefs about organ donation and transplantation did not always apply to a specific ‘mode’ of transplantation and were inextricably linked or related to higher level beliefs.”* |
| 2 | The use of the word “UK Chinese” or UK Chinese individuals/people/men/women” seems a bit out of ordinary and perhaps may be politically incorrect especially given the fact that only 4/16 were born in Mainland China. May be acceptable. I am unsure as I do not belong to this community. I would confirm with a native individual how do would like to be addressed in scientific literature. Also, I would read the paper in JAMA that made recommendations on how to report race and ethnicity | We thank Reviewer 1 for querying the use of this descriptive term. One of our co-authors identifies as belonging to this group and has used this term in other published work in PLOS ONE [<https://pmc.ncbi.nlm.nih.gov/articles/PMC8884499/> , <https://pubmed.ncbi.nlm.nih.gov/36649253/>]. This term was used in all communications with representative organisations and potential participants, meaning all individuals recruited self-identified as belonging to this group. We believe that the term UK Chinese aligns with the guidance on reporting race and ethnicity in the article mentioned by Reviewer 1. This article recommends that:  “For studies that use national databases or include participants in a single country, a term for country of origin can be included if the term was provided at data collection (e.g., Chinese American and Korean American for a study performed in the US, or Han Chinese and Zhuang Chinese for a study conducted in China). Again, how these designations were determined (e.g., self-reported or selected or by other means) should be reported.”  We have clarified who is included in our study with the following information in the methods at line 72-75.  *‘In this qualitative interview study, we undertook in-depth semi-structured interviews with individuals aged 18 and over who self-identified as UK Chinese.* *UK Chinese included people who self-identified as being of Chinese heritage who were currently resident in the UK. It included people born in the UK or outside the UK, having subsequently emigrated.’* |
| 3 | The team incorrectly uses the terminology for gender. It should be woman and man, not male and female | We have changed male/female to man/woman. |
| 4 | The discussion is also quite lengthy and I instead would suggest making it more succinct, and perhaps consider expanding on the results. | We have made the discussion more succinct throughout, reducing the overall word count. |
| 5 | This is also an educated group of participants, most of whom spoke English, hence I would suggest addressing a potential sampling/representative bias | We aimed to achieve a diverse sample, rather than a population representative sample which is associated with quantitative research. Qualitative studies seek to provide in-depth and diverse insights into a phenomenon and do not attempt to generalise the results to a population of interest [Reference <https://pubmed.ncbi.nlm.nih.gov/10891968/>]. Most qualitative studies are necessarily small scale to provide depth and contextualised detailed information, and thus the criterion of generalisability (external validity) cannot be applied. Instead, transferability is the extent to which the concepts and theories are relevant to other settings. Researchers can compare their results with studies conducted in different healthcare contexts, regions, or populations; position their findings with other theoretical frameworks; and describe the study setting and participant characteristics in detail so readers can judge the transferability of the findings to their own context [Reference <https://journals.lww.com/transplantjournal/Fulltext/2016/04000/Qualitative_Research_in_Transplantation__Ensuring.9.aspx>].    Participants in our study were diverse in terms of age, gender, place of birth, year of emigration to the UK (if applicable) and education level. In the 2021 UK Census, only 1.8% of the UK population could not speak English. 2/16 (13%) participants in our study were interviewed with a Cantonese interpreter. We would highlight that 7/16 participants’ highest level of education was school or vocational training, not a university undergraduate or post-graduate education, indicating that not all participants were highly educated, again representing diversity.  The UK Chinese population is a highly educated group: pupils from the Chinese ethnic group have the highest entry rate into higher education out of all ethnic groups in the UK (Reference <https://www.ethnicity-facts-figures.service.gov.uk/summaries/chinese-ethnic-group/>) and we have added a sentence to our discussion at line 371-373 to describe this.  *“This was not identified in our study; however, may be explained by 9/16 participants having university-level education, which broadly reflects the high educational attainment amongst UK Chinese compared to other UK ethnic groups [16].”* |
|  | **Reviewer 2** |  |
| 6 | The authors could clarify the methods to analyze data from translated interviews. The methods mention KW verified translations. After this step, was participant commentary no longer used and transcriptions and coding used only translator text (e.g. only English was transcribed)? | Reviewer 2 correctly describes our approach, where translators were used, analysis was based on transcription of the interpreter’s speech in English. We have added an additional sentence to our methods at lines 107-108 to make this clearer.  *“When interpreters were used, only the interpreter’s speech in English was transcribed; and analysis thereafter was of English transcriptions only.”* |
| 7 | The topic guide includes a question about personal relationships impacted by kidney disease, but this is not captured in the results from themes or questionnaires. It is unclear if this question was coded. Some quotes suggest detailed knowledge of kidney disease and other quotes suggest significant misunderstandings. Information about the sample’s familiarity with the topic would be relevant. | Data were collected through semi-structured interviews, not structured interviews or questionnaires. The flexible topic guide is designed to indicate a series of potential topics to discuss: the questions listed are not asked verbatim and the guide evolves as interviews progress. In thematic analysis as per Braun and Clarke, coding is not undertaken according to each ‘question’ or topic, but rather coding is inductive, and data driven. Initial codes were generated by assigning descriptive labels to interesting features of the data and sections of text.  Reviewer 2 is correct that participants appeared to have a varying degree of familiarity with the subject matter, further evidencing that we achieved a diverse sample as intended. Some respondents provided detailed descriptions of experiences of family/friends, while others appeared to have little direct exposure. We have described this in the following section which we have added at line 132-137.  *“Our diverse sample included individuals who had experience of a friend or family member living with kidney disease and individuals who had not been personally exposed to kidney disease or its treatments. No participants reported living personally with kidney disease. We did not identify a clear relationship between the degree of familiarity with the subject matter and the themes that emerged: for example, those who had some awareness of a person’s lived experience did not appear more supportive of organ donation and transplantation than those without this experience.”* |
| 8 | The sample quotes provide a rich qualitative foundation, but some themes and subthemes seem better supported than others. Negative impacts on donor health theme is complicated by potential exaggerated perceptions of donor impacts, and this may be a relevant distinction. In addition, quotes related to fear may not be specifically related to donor health as much as the acute experience. The description of this theme also reinforces the concept of abstract suffering, suggesting a theme label that is broader than “donor health” may be appropriate. | Reviewer 2 is correct that participants appeared to have varied concerns about impacts of donation on donors, referencing both short- and long-term effects. These concerns all broadly related to donor health, including the notion of ‘suffering’ which appeared to reflect worries about physical pain. To reflect the breadth of responses we have changed the title of the theme to read “Negative impacts on donors”. We have edited the results section to describe whether concerns were focussed on short term or long-term impacts and to what extent these concerns were with reference to health (medical/surgical/psychosocial).  The results section now reads as follows:  ***“Negative impact on donors.*** *Many participants expressed concerns about potential negative short- and long-term effects on living kidney donors. For example,* *several participants expressed the view that LKD negatively impacts the long-term health of a donor. Some ascribed significance to having “two kidneys for a reason”, although one participant stated that “we can rely on one”. One participant described how donating a kidney would constitute “purposefully making you ill” resulting in “long-term health complications”. No participant stated explicitly that they were worried about the increased risk of kidney failure, however reference to the importance of having two kidneys did seem to imply that participants were concerned about the impact of donation on kidney function. Many participants described concerns about physical pain or suffering in the immediate aftermath of major surgery. One participant described how this was not “just an ethnic community thing” but something relevant to individuals more broadly.”* |
| 9 | The Passivity theme is most difficult to interpret. The description implies that since support for transplantation was only voiced in terms of what others could do and not what they could do, that participants were passive. This is somewhat contradicted by quotes that participants would certainly donate to family if asked. A potential limitation of this theme is that the topic guide does not seem to have questions that would reasonably elicit a meaningful response for the extent that the participant was passive. A question asks where information could be provided. If Quote #3 (e.g. discussion of leaflets in supermarkets) was in response to this type of question, it is potentially misleading to assign a meaning related to willingness to personally act or not act. | Data were collected through semi-structured interviews, not structured interviews or questionnaires. The flexible topic guide is designed to indicate a series of potential topics to discuss: the questions listed are not asked verbatim and the guide evolves as interviews progress. In thematic analysis as per Braun and Clarke, coding is not undertaken according to each ‘question’ or topic, but rather coding is inductive, and data driven. Initial codes were generated by assigning descriptive labels to interesting features of the data and sections of text.  We thank Reviewer 2 for their suggestion to revisit our presentation of ‘passivity’. We have relabelled the sub-theme of passivity as ‘a perceived need for education and engagement’ to better reflect the content of this theme. The intention of this theme was to capture the disconnect between the recognition amongst participants that greater awareness and education about transplantation would be beneficial for increasing access to transplantation, coupled with passivity about seeking out this information. We believe that the way participants described accessing further information implied there were other factors influencing individual behaviours. We have now rewritten this sub-theme to read as follows:  ***“A perceived need for education and engagement.*** *Several participants stated that greater access to information was needed to improve willingness to engage in organ donation. Some respondents described the importance of a small number of ‘highly trusted’ institutions in providing information, often directly referencing the UK National Health Service. Despite this perceived need for greater knowledge and awareness at a population level, no-one described an intention to improve their personal understanding of organ donation. In an acknowledgement of this pattern of behaviour, one participant described their enthusiasm for the ‘opt-out’ law in terms of its ability to widen access to transplantation in an environment where individuals fail to seek information and/or formalise their views. The disconnect between participants expressed views and their own behaviours seemed to imply other factors were influencing their willingness to seek out information, although these were not explicitly described by participants. Hesitance to discuss issues relating to organ donation with family, and a broader culture of silence, may both have been relevant and are described in detail above. In addition, the emphasis on ‘trust’ implied participants were concerned about this risk of misinformation.”* |
| 10 | There is a typo on p 26, line 285. | We have now corrected this. |
| 11 | The discussion includes a valuable review of related literature and how current findings compare to the body of research. The authors cite studies of Chinese Canadians; however, several more recent studies of South Asian and Chinese Canadians populations could provide additional insight. | We are not entirely clear to which recent studies Reviewer 2 is referring. We have conducted a review of relevant literature and were unable to find many studies that provided significant additional insight. We have added a reference to a survey of Asian Americans (a proportion of which were Chinese Americans) reporting how infrequently conversations about organ donation take place (<https://pmc.ncbi.nlm.nih.gov/articles/PMC11315553/>). The majority of studies of ‘Asian’ populations in American include South Asians, East Asians, Southeast Asians, and Central Asians, and only a minority of Chinese Americans. |
| 12 | The discussion touches on the potential tension between Familism and Culture of Silence, but this seems under explored and potentially of interest. This seemingly describes a perception that donation by others to family is a sacrifice more than a duty but a donation by oneself to family is more a duty and less a sacrifice. While this may be a poor articulation as a western reader, it would be a potential issue to address further. | We thank Reviewer 2 for drawing this lack of clarity to our attention. We agree this is an important point and have now expanded on this concept in our discussion. Lines 353-367 now read:  *“Participants in our study suggested family members would likely feel duty-bound to help others in their family through organ donation. Participants indicated they would be reluctant to initiate conversations with family about donation if they were suffering from kidney disease, so as not to impose this duty on others, particularly given organ donation was seen as negatively impacting donors. A similar concern among potential organ recipients has been reported in a retrospective analysis of 1273 potential kidney recipients in South Korea, where worries about the health of the potential donor were the most common recipient-related reason for discontinuation of the LKD process [39]. The ‘culture of silence’ extended to conversations around deceased kidney donation, suggesting concerns about imposing a ‘duty to donate’ on family members was not the only reason limiting open discussions, reinforcing the notion that reticence is culturally engrained at a deeper level. Consistent with this finding, UK transplant data indicate that uncertainty about whether a patient would have agreed to donation was the second most common reason for families of UK-Chinese individuals to decline consent to deceased kidney donation [27]. By contrast, families of South Asian and Black donors were more likely to state that a relative had previously expressed a wish not to donate, suggesting a prior discussion had taken place [27].”* |
|  | Editor |  |
| 13 | How the term UK-Chineese affected the eligebility for the study | We thank the editor for their comment and direct them to our previous response (#2), which addresses a similar point. |
| 14 | It would be intersting to have some data of other UK population, if possible to compare with (Indian or African). Authors may add some data especially if those works used simillar methodology. | Where possible we have attempted to compare our findings with prior similar research in UK ethnic minority groups.  For example, when discussing the importance of bodily integrity, we compare our results with similar qualitative work in the Black British Caribbean community (lines 301-304). We also consider how a ‘culture of silence’ may impact UK minority ethnic groups differently, referencing survey data that includes UK South Asian and Black respondents (lines 362-367). |
